# Interventions for adolescents and adults with psychosis in Africa: a systematic review and narrative synthesis

**DOI:** 10.1017/gmh.2022.25

**Published:** 2022-05-27

**Authors:** Xanthe Hunt, Haleem Abdurahman, Olubukola Omobowale, Adeola Afolayan, Epiphania Munetsi, Lloyd Dzapasi, Nyareso Mokaya, Alhaji Koroma, Ibrahim Barrie, Olusegun Ogunmola, Abubakar Koroma, Tom Shakespeare, Julian Eaton, Grace Ryan

**Affiliations:** 1 Institute for Life Course Health Research, Department of Global Health, Stellenbosch University, Stellenbosch, South Africa; 2 Department of Child and Adolescent Psychiatry, University College Hospital, Ibadan, Nigeria; 3 Centre for Child and Adolescent Mental Health, College of Medicine, University of Ibadan, Ibadan, Nigeria; 4 Department of Community Medicine, College of Medicine, University of Ibadan, Ibadan, Nigeria; 5 Research Support Centre, University of Zimbabwe, Harare, Zimbabwe; 6 London School of Hygiene & Tropical Medicine, London, UK; 7 University of Makeni, Makeni, Sierra Leone; 8 International Centre for Evidence in Disability, London School of Hygiene & Tropical Medicine, London, UK; 9 CBM Global Disability Inclusion, Laudenbach, Germany; 10 Centre for Global Mental Health, London School of Hygiene & Tropical Medicine, London, UK

**Keywords:** Africa, psychosis, schizophrenia, systematic review

## Abstract

**Background:**

The Global Burden of Disease attributable to psychotic disorders in African countries is high and has increased sharply in recent years. Yet, there is a scarcity of evidence on effective, appropriate and acceptable interventions for schizophrenia and other psychotic disorders on the continent.

**Methods:**

We carried out a systematic review and narrative synthesis of peer-reviewed literature evaluating the impact of non-pharmacological interventions for adolescents and adults (10–65 years) in African countries. Two reviewers independently double-screened all articles and performed data extraction and quality appraisal using standardized tools.

**Results:**

Of the 8529 unique texts returned by our search, 12 studies were identified for inclusion, from seven countries: Egypt, Ethiopia, Ghana, Kenya, Nigeria, South Africa and Sudan. They evaluated a range of interventions with one or more clinical, psychological or psychosocial, education or awareness or traditional or faith-based components, and were delivered by either mental health specialists or non-specialist health workers. Ten of the 12 included studies reported significant, positive effects on a range of outcomes (including functioning, symptoms and stigma). Nearly half of the interventions were based out of health facilities. Based on quality appraisals, confidence in these studies' findings is only rated low to medium.

**Conclusion:**

Further research is needed to develop and evaluate interventions that meet the diverse needs of people with psychosis, within and beyond the health sector.

## Background

Psychosis refers to a range of symptoms, including hallucinations, delusions and disorganized thinking (Cooke, 2017), which characterize several mental health conditions, such as schizophrenia, schizotypal and delusional disorders (WHO, 1993). Although these conditions are estimated to affect only about 1% of the global population (Moreno-Küstner *et al*., 2018), they are among the most severely disabling (Vos *et al*., 2017; Charlson *et al*., 2018). For instance, in the Global Burden of Disease (GBD) studies, acute schizophrenia (schizophrenia in the active phase) has the highest disability weight of any physical or mental health condition (Salomon *et al*., 2015). As a result, schizophrenia contributes more than 12 million disability-adjusted life years to the GBD, despite its relatively low prevalence (He *et al*., 2020). Population growth and ageing have driven a substantial increase in the GBD attributable to psychotic disorders, particularly in low- and middle-income countries (LMICs) (Charlson *et al*., 2018). Yet, there is a scarcity of evidence on effective, culturally appropriate and acceptable interventions for schizophrenia and other psychotic disorders in ‘non-Western’ settings to guide decision-making by LMIC governments and programme managers (Degnan *et al*., 2018).

There is an urgent need to increase access to high-quality mental health care for people with psychosis in the African region. It has the fewest mental health workers (0.9 per 100 000 population), mental health beds (2.5 per 100 000) and outpatient facilities (0.07 per 100 000) of any world region, and in 43% of African countries, service users pay mostly or entirely out of pocket for treatment (WHO, 2018). In part due to resource limitations, the proportion of people with psychosis who receive treatment is low, even when compared to other LMICs around the world (Lora *et al*., 2012; Fekadu *et al*., 2019). Furthermore, the treatment received may not meet the diverse needs of people with psychosis. A cross-sectional survey from a rural district of Ethiopia found that of the approximately 40% of people with psychosis who had received treatment for a current episode, more than 70% did not receive ‘minimally adequate care’ (defined for the purposes of the study as four or more visits of follow-up and medication monitoring), despite living near a well-established mental health research site (Fekadu *et al*., 2019). Reports of polypharmacy (Ayenew *et al*., 2021) and other potentially harmful or abusive practices – such as restraint and seclusion – are common, including in psychiatric facilities (HRW, 2012; MDAC and MHU, 2014; MDAC, 2017; HRW, 2019), and the availability of psychological, psychosocial and other non-pharmacological interventions is limited (Patel *et al*., 2011; Brooke-Sumner *et al*., 2015). Likewise, integrated models of care like community-based rehabilitation (CBR), which aim to make a range of different supports available at the community level, are relatively scarce (although their presence is growing in LMICs) (Brooke-Sumner *et al*., 2015).

Despite these challenges, the African region has also been a driver of research and innovation in mental health (Qureshi and Eaton, 2020). Nearly half (40%) of recent peer-reviewed studies documenting the application of the World Health Organization's (WHO) mental health Gap Action Program intervention guide (mhGAP-IG) come from Africa – more than any other world region (Keynejad *et al*., 2021). Several of the largest international non-governmental organizations working in global mental health have flagship projects in Africa (e.g. The Carter Center, CBM Global, Partners in Health). International funders like the United Kingdom's Foreign Commonwealth and Development Office (formerly the Department for International Development) (Lund *et al*., 2012), Grand Challenges Canada (Kisa *et al*., 2016) and the European Commission (Puschner *et al*., 2019) have also invested in research consortia evaluating interventions for schizophrenia and other severe mental health conditions in African countries.

Yet, relatively few African studies have been identified by previous reviews of interventions for psychotic disorders in LMICs (De Silva *et al*., 2013; Brooke-Sumner *et al*., 2015; Asher *et al*., 2017; Demissie *et al*., 2018). This review uses broad inclusion criteria to describe the scope and nature of interventions which have been tested for people with psychosis in Africa, and to assess where there is evidence of impact.

It is worth noting that while the authors adhere to a disability rights-informed perspective on mental health and psychosocial disability, the literature covered in this systematic review derives from a variety of fields and perspectives, and so the language used throughout reflects both terminologies. We have sought to adhere to the language used in the individual publications where relevant.

## Methods

A review protocol was developed in accordance with the Preferred Reporting Items for Systematic Reviews and Meta-Analyses Protocol (PRISMA-P) guidelines through a participatory process and registered with the International Prospective Register of Systematic Reviews (PROSPERO; CRD42020212873). A 15-member Support, Comprehensive Care and Empowerment of People with Psychosocial Disabilities in Sub-Saharan Africa (SUCCEED) advisory group comprised of experts by lived experience and experts by professional experience (clinical and/or research) from seven countries (Kenya, Nigeria, Sierra Leone, South Africa, Zimbabwe, United Kingdom and United States) was convened at the early stages of protocol development to advise on methodological decisions. SUCCEED is a Health Research Programme Consortium, and results of this review will directly inform the development and methods of evaluation of a complex intervention for people with psychosis in West and Southeast Africa as part of SUCCEED's programme of research. Advisory group members were invited to contribute to each stage of the review's conduct, from screening through data extraction, and were provided with relevant training by the first author, as needed. Three additional advisory group meetings were held to review early findings and contribute to the interpretation of results. Further details on the review methodology are provided below.

### Eligibility criteria

Our review included peer-reviewed, published literature concerning published research studies evaluating interventions for people with psychosis in Africa, including both adults and adolescents.

#### Population

We included studies which examined the impact of interventions for adolescents and/or adults who have a current or previous history of psychotic disorders or symptoms. We intended to exclude any studies in which the mean participant age was below 10 or above 65 years, as psychosis is exceedingly rare in children, and it is common for elderly people with dementia to be misdiagnosed and/or treated with antipsychotic medications in African settings (Truter, 2013). However, this proved to be unnecessary, as we found no studies in which the mean participant age was outside this range.

#### Interventions

We were interested in assessing the full spectrum of interventions targeting the population of interest, with the exception of purely pharmacological treatment, including, but not limited to psychological interventions, social protection interventions, health interventions, livelihoods interventions, education interventions, life skills interventions and social inclusion and empowerment interventions. We excluded studies assessing the efficacy and effectiveness of medication alone (i.e. drug trials), or where the focus of the programme was on a medication intervention (even if there were adherence monitoring components), as this evidence has recently been synthesized for the revision of mhGAP-IG in 2015 (WHO, 2015) and a systematic review published in 2020 (Kumar *et al*., 2020). We did not place any restriction on the type of provider delivering the programme.

#### Comparators

We placed no restrictions on the comparator/control groups against which interventions of interest were compared, and we did not insist that studies include a comparator or control group, although our quality appraisal criteria did evaluate studies based on whether they were controlled.

#### Types of studies

Eligible studies were those that were designed to assess intervention impact (including, for instance, randomized controlled trials, controlled and uncontrolled before and after designs). Descriptive studies such as cross-sectional interview studies and single time point surveys were not included.

#### Setting

We limited the scope of our search geographically to the African continent. We included countries from World Bank lists of Sub-Saharan African (World Bank, n.d.-*b*) and North African countries (World Bank, n.d.-*a*).

#### Outcomes

We included individual outcomes for people with psychosis. We excluded service- and system-level outcomes, as these are outside the scope of this review. Where studies included populations of people with multiple diagnoses, outcomes needed to be disaggregated for people with psychoses in order for a study to be eligible.

### Search strategy

We employed terms related to the population (people with psychosis) and locations (African countries). We tailored the search strategy for each database and exploded subject headings where relevant. The search strategy can be seen in the Supplementary materials. We searched CINAHL, ERIC, Scopus, Web of Science (Social Sciences Citation Index), MEDLINE, PubMed, Embase Classic + Embase, PsycINFO and CABI Global Health on 14 October 2020. No restrictions in terms of date or format were placed on the search, but only English-language publications were eligible (due to limitations of the review team).

### Selection process

**Fig. 1.**
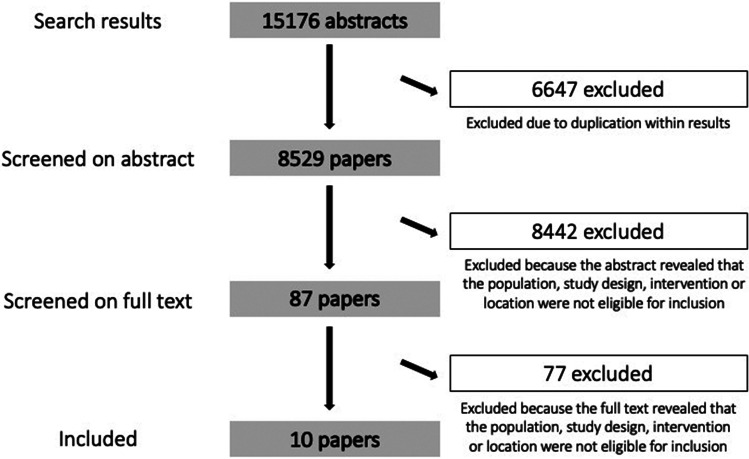
PRISMA flow chart. Detailed reasons for exclusions of studies are available from the authors upon request.

We used Microsoft Excel (Microsoft, 2019) for bibliographic management, screening, coding and data synthesis. We screened all unique references from our search on title and abstract, with two independent reviewers determining relevance of each study. If any disagreement arose, it was resolved by the first (XH) and/or last (GR) author. A similar process was followed for full text screening. The screening process is reported using a PRISMA flow chart (see Fig. 1; reasons for exclusion can be obtained from the study authors upon request).

### Data collection process and data items

Two independent reviewers coded the included studies. They extracted data from the studies according to a coding sheet which was developed by the first (XH) and last authors (GR) and then refined through consultation with the advisory group. All coding sheets were checked by the first author. Studies were coded by intervention, outcomes and a range of other characteristics, such as age of target population and method of intervention delivery.

### Risk of bias (confidence in study findings) assessment

Two independent researchers rated each included study according to a pre-determined tool adapted from Saran *et al*. (2020). Confidence in study findings was rated as high, medium or low, for each of the following six criteria: study design, masking, attrition, clear definition of psychosis, clear definition of outcome measures and baseline balance. Overall confidence in study findings was recorded as the lowest rating a study achieves across the criteria.

### Effect measures

Due to the heterogeneity of interventions, outcomes and outcome measures, we did not calculate standardized effect sizes or perform a meta-analysis. Author-reported effect sizes and *p* values were extracted and are noted in our Results section (see Table 1).

**Table 1.** Summary of findings

		Intervention	Country	Study design	Sample size	Inclusion criteria	Outcome/s	Summary findings
Included studies	Engelbrecht *et al*. (2019)	Occupational therapy day clinic	South Africa	Uncontrolled before *v.* after	44	Adolescents and adults receiving routine care at day clinic. DSM-IV diagnostic criteria used.	Admissions	33 fewer admissions (62.3%) over 24 months post-intervention (*z* = −4.093, *p* = 0.00)
							Number of days spent in hospital	A reduction of 74.5% (2569 days) over 24 months post-intervention (*z* = −4.730, *p* = 0.00)
	De Menil *et al*. (2015)	Community-based intervention delivering medical care and self-help groups	Kenya	Uncontrolled before *v.* after and cost analysis	117	Adults with a diagnosis of psychosis. Diagnostic checklist not specified.	Symptoms/functioning (GHQ-12)	There were decreases in GHQ-12 of 4.2 points (SMD) *p* = 0.000 in the first year and 8.3 (SMD) *p* = 0.00 over two years.
	Gohar *et al*. (2013)	Social cognitive skills training	Egypt	RCT	42	Adults presenting to a psychiatric hospital. Structured Clinical Interview for DSM-IV disorders (SCID) criteria for schizophrenia or schizoaffective disorder used.	Social cognition (MSCEIT)	The intervention group demonstrated significant treatment effects on total emotional intelligence scores (*F* = 24.31, *p* < 0.001), compared to control.
							Identifying emotions (MSCEIT subscale)	The intervention group demonstrated significant treatment effects on identifying emotions (*F* = 11.77, *p* < 0.001) compared to control
							Using emotions (MSCEIT subscale)	There were no significant effects on using emotions post-intervention (*F* = 1.21, *p* > 0.05)
							Understanding emotions (MSCEIT subscale)	There were no significant treatment effects for understanding emotions (*F* = 0.29, *p* > 0.05)
							Managing emotions (MSCEIT subscale)	There were significant effects for managing emotions (*F* = 23.27, *p* < 0.001),
							Neurocognition (Trail Making Test Part A; Digit Symbol Substitution Test; Digit span task Proteus Mazes task)	There were no significant effects for neurocognition (*F* = 0.03, *p* > 0.05)
							Symptoms/functioning (PANSS)	There were no significant effects for PANSS (*F* = 0.01, *p* > 0.05)
	Brooke-Sumner *et al*. (2018)	Non-specialist delivered psychosocial rehabilitation	South Africa	Uncontrolled before *v.* after	44	Adults with a diagnosis of psychosis. Diagnostic checklist not specified.	Symptoms/functioning (WHODAS)	A non-significant reduction in WHODAS total scores from baseline to endline over 12 months. Median difference of −2.8, *z* = 0.92, *p* = 0.358.
							Symptoms/functioning (BPRS)	A non-significant reduction in BPRS total scores from baseline to endline over 12 months. Median difference of −3, *z* = 0.77, *p* = 0.442.
							Stigma (ISMI)	A significant reduction in ISMI total scores from baseline to endline over 12 months. Median difference of −2, *z* = 2.04, *p* = 0.041.
							Unemployment	Unemployment reduced from 95.7% at baseline to 81.4% at endline over 12 months (χ^2^ = 4.68, *p* = 0.044).
	Sibeko *et al*. (2017)	Treatment-partners, psychoeducation,	South Africa	RCT	77	Adults with a diagnosis of psychosis. Structured clinical interview for	Symptoms/functioning (PANSS)	There were no significant differences in PANSS scores, aMD = −13.1, *p* = 0.062 based on an ITT analysis.
		and clinic appointment reminders				diagnosis of axis-I disorders (SCID-I) used.	Medication adherence (MARS)	There were no significant differences in MARS scores aMD = 0.49, *p* = 0.603 based on an ITT analysis.
	Rami *et al*. (2018)	Adapted version of the Behavioural Family Psycho-Education Program (BFPEP)	Egypt	RCT	60	Adults meeting DSM-IV criteria for schizophrenia.	Symptoms/functioning (PANSS)	PANSS scores decreased significantly for the intervention group post-intervention *t* = 6.1, *p* < 0.01.
							Medication attitudes (DAI)	DAI scores increased significantly for the intervention group post-intervention *t* = −6.3, *p* < 0.01.
							Quality of life (QLS)	QLS total scores increased significantly for the intervention group post-intervention *t* = 5.7, *p* > 0.05.
							Symptoms/functioning (SFQ)	SFQ scores increased significantly for the intervention group post-intervention *t* = −6.7, *p* < 0.01.
	Gureje *et al*. (2020)	Manualized collaborative care by traditional and faith healers and primary healthcare workers	Ghana and Nigeria	RCT	307	Adults with psychosis who were not actively symptomatic at the time of recruitment. Structured clinical interview for diagnosis of axis-I disorders (SCID-I) used.	Patient exposure to harmful practices	Harmful practices decreased from 94 (57% of patients) to 13 (9% of patients) at 6 months in the intervention group (−0.48 aMD, *p* < 0.001) and from 59 (42% of patients) to 13 (10%) in the control group (−0.33 aMD, *p* < 0.001). There was no significant difference between the two groups.
							Symptoms/functioning (PANSS)	PANSS total mean scores at 6 months decreased significantly, aMD −15.01, *p* = 0.0001.
							Stigma (ISMI)	ISMI total mean scores over the 6 month trial-period decreased significantly, aMD −0.2 *p* = 0.013
							Symptoms/functioning (WHODAS)	WHO DAS total mean scores over the 6-month trial-period decreased significantly, aMD −10⋅5, *p* = 0.0015
							Course of illness and recovery	Months on admission aMD = −0.7, *p* = 0.029; Course of illness aMD = 2.5 *p* = 0.0032; Engagement in work aMD = 3.3 *p* = 0.0003; Ever in independent living aMD = 2.4 *p* = 0.27.
							Victimization	The proportions of participants reporting having experienced victimization of any type over the 6-month trial period were similar in the intervention and control groups adjusted odds ratio 0.80; *p* = 0.70).
							Cost effectiveness	At 6-month follow-up, the intervention was both more effective and less costly than the control, cost associated with a one-point improvement on the PANSS was –$4 (95% CI −29 to 15) and –$4 (−29 to 18) on WHO-DAS in the intervention group.
	Thomas *et al*. (2017)	SMS text reminders of clinic appointments	Nigeria	RCT	200	Adults with psychosis. ICD-10 diagnostic criteria used.	Appointment attendance	Receiving an SMS almost doubled the likelihood of attendance in the intervention group compared to the control [odds ratio (OR) = 1.80, *p* = 0.001]
							Missing appointment	Receiving an SMS reminder decreased the odds of missing their next appointment OR = 0.5, *p* < 0.03
	Asher *et al*. (2018)	Community-based rehabilitation attached to task-shared mental health care	Ethiopia	Uncontrolled before *v.* after	Quantitative: 10 people with psychosis, each with a family caregiver (*n* = 20)	Adults with a diagnosis of schizophrenia spectrum disorder	WHODAS	Change in baseline median WHODAS score from 57.5 [IQR (interquartile range) 36.7, 65.1], to 18.4 (IQR 2.4, 46.2) 12 months post-intervention.
						(schizophrenia, schizoaffective disorder or)	Clinical global impression (% normal/borderline)	change from 0% at baseline to 50% at 6 months and 62.5% at endline
						(schizophreniform disorder) using DSM-IV criteria	Discrimination (DISC-12 total)	median 2 (IQR 0,4) at baseline, to median 0 (IQR 0,4) at 6 months, and median 0 (IQR 0,3.5) at endline.
						(assessed using the Operational Criteria for)	Depression (PHQ9 total)	10.5 (6,13) at baseline, to 6 (2,11) at 6 months, and 3.5 (1.5,8.5) at endline
						Research (OPCRIT).	Alcohol use (AUDIT total)	increased initially from 3.5 (0,7) at baseline to 4.5 (2,13) at 6 months and then dropped to 2.5 (0,5.5) at endline
							Caregiver burden (IEQ)	decreased from 46 (37,61) at baseline to 26.5 (21,48) at 6 months before increasing slightly (but not to pre-baseline levels) at endline 30.5 (19,41.5)
	Hanlon *et al*. (2020)	District-level plan for task-shared mental health care	Ethiopia	Uncontrolled before *v.* after	300	Adults with psychosis. Operational CRITeria for research (OPCRIT) used.	Clinical and social severity	There was a significant improvement in all clinical (symptom severity score, depressive symptoms, suicide attempts, alcohol use disorder) and social (functioning, discrimination, restraint) outcomes of 0.28 *p* = 0.0004 over 12 months post-intervention, measured as the standardized mean difference.
							Disability	The impact on disability (standardized mean difference) was 0.50; *p* = 0.00 which was greater than the impact on symptom severity over 12 months post-intervention.

### Synthesis methods

Our approach to data synthesis was a narrative synthesis, a method suited for systematic reviews with heterogeneous studies in terms of study design, interventions and outcomes. Drawing on the guidance from Popay and colleagues (Popay *et al*., 2006), our analysis involved:

1. developing a preliminary synthesis of findings of included studies;
2. exploring relationships in the data;
3. assessing the robustness of the synthesis.

## Results

Our search yielded 15 176 abstracts, of which 6647 were duplicates. The remaining 8529 papers were screened by title and abstract by a team of 12 reviewers, working in pairs. Each title and abstract was screened by both pair members, independently. Based on this process, 8442 papers were excluded as their abstract did not indicate that the associated study met inclusion criteria. Six reviewers, working in pairs, screened the remaining 87 full texts independently. A further 77 studies were excluded because an examination of the full text revealed that the study did not meet inclusion criteria. This resulted in the final set of 10 papers identified for inclusion (Fig. 1).

### Included studies

Three studies came from South Africa, two from Ethiopia, one from Kenya, two from Egypt and one from Nigeria alone. An additional multi-site study was carried out in both Ghana and Nigeria. Of the included studies, five used an uncontrolled before *v.* after design, and five were randomized controlled trials.

Table 1 presents a summary of the included studies.

### Confidence in study findings

Our confidence in the overall findings is low to medium, on the basis of our study appraisals (see Table 2).

**Table 2.** Quality appraisal

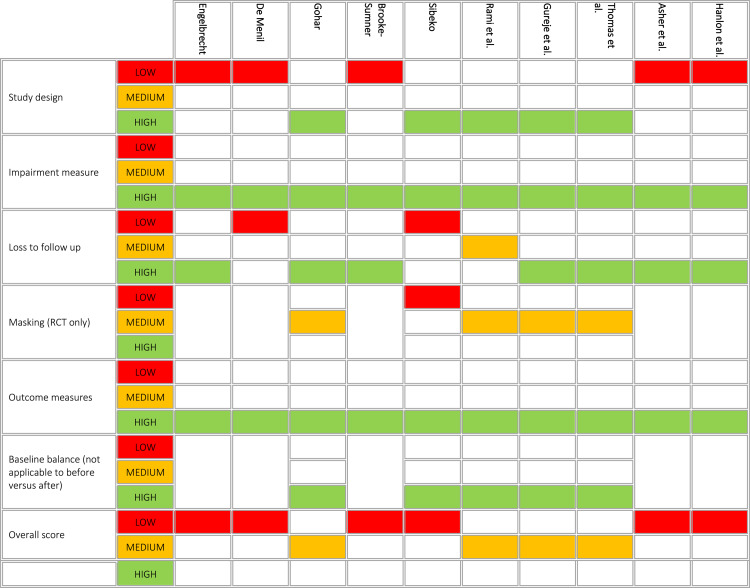

Only four studies (Gohar *et al*., 2013; Thomas *et al*., 2017; Rami *et al*., 2018; Gureje *et al*., 2020) scored medium using our assessment tool, with the remaining six (de Menil *et al*., 2015; Sibeko *et al*., 2017; Asher *et al*., 2018; Brooke-Sumner *et al*., 2018; Engelbrecht *et al*., 2019; Hanlon *et al*., 2020) scoring low. Low ratings were largely due to studies employing uncontrolled or controlled before *v.* after designs (de Menil *et al*., 2015; Asher *et al*., 2018; Brooke-Sumner *et al*., 2018; Engelbrecht *et al*., 2019; Hanlon *et al*., 2020).

#### Narrative synthesis

Our narrative synthesis identifies trends among the included studies, across three key domains: the content of the interventions employed, how and in what contexts they are delivered, the outcomes they target and intervention impact as detailed in Tables 3–5.

**Table 3.** Details on intervention content, by country

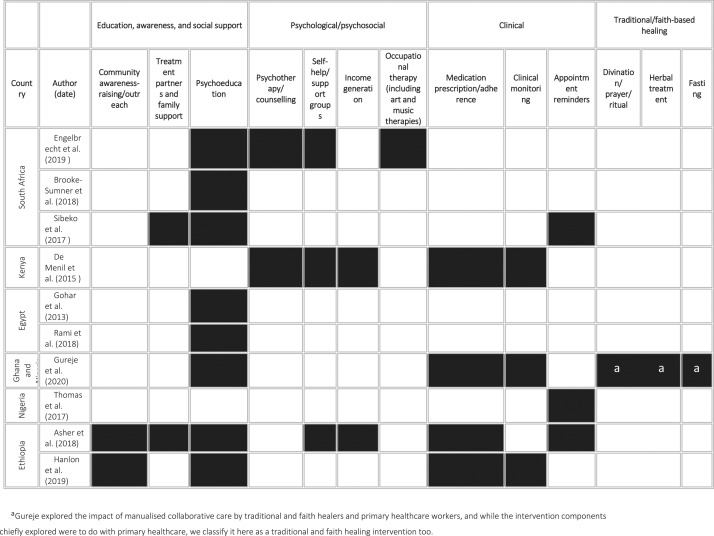

**Table 4.** Details on delivery of psychosis interventions, by country

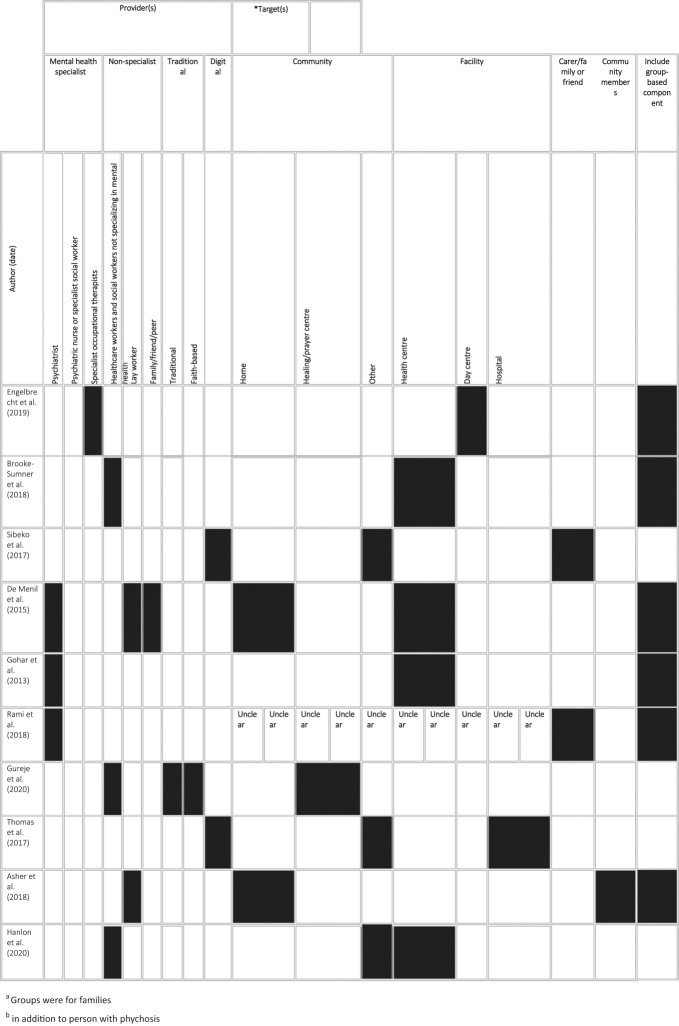

**Table 5.** Quantitative outcomes and measures

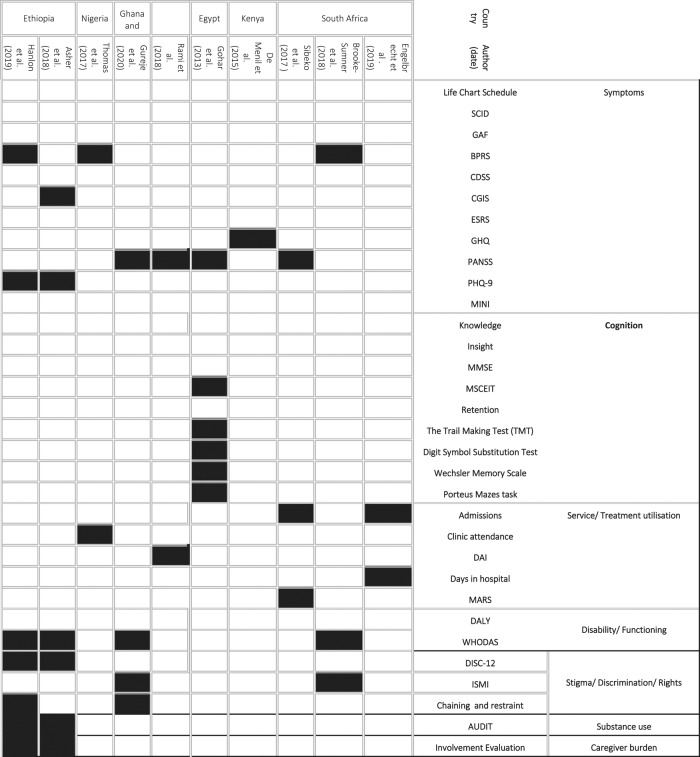

*Intervention content*. Key components of the included interventions are presented by country in Table 3. Although we found very few countries with more than one study, it is interesting to note some possible trends. In Egypt, both studies examined psychoeducation interventions. In Ethiopia, the two studies were related, with a pilot CBR intervention evaluated in one study attached to the implementation of task-shared mental health care evaluated in another. South Africa had the most studies and the most diverse interventions, with several involving psychoeducation and psychological or other psychosocial approaches.

The majority of interventions fell into the category of education, awareness and social support. This included programmes engaging in community awareness-raising and outreach, those involving treatment partners and family support and those delivering psychoeducation. Indeed, eight interventions (Gohar *et al*., 2013; Sibeko *et al*., 2017; Asher *et al*., 2018; Brooke-Sumner *et al*., 2018; Rami *et al*., 2018; Engelbrecht *et al*., 2019; Gureje *et al*., 2020; Hanlon *et al*., 2020) included psychoeducation components, making it the most common form of intervention content by far. Only two studies involved treatment partners and family support (Sibeko *et al*., 2017; Asher *et al*., 2018), and two included community awareness-raising and outreach (Asher *et al*., 2018; Hanlon *et al*., 2020). The second most common category of intervention was clinical, comprising medication prescription or adherence support, clinical monitoring and appointment reminders. Four studies (de Menil *et al*., 2015; Asher *et al*., 2018; Gureje *et al*., 2020; Hanlon *et al*., 2020) provided medication prescription or adherence support, and another three (de Menil *et al*., 2015; Gureje *et al*., 2020; Hanlon *et al*., 2020) included clinical monitoring. Appointment reminders were delivered as part of three (Sibeko *et al*., 2017; Thomas *et al*., 2017; Asher *et al*., 2018) interventions.

Psychological and/or psychosocial support was the third most common. Three interventions involved self-help or support groups (de Menil *et al*., 2015; Asher *et al*., 2018; Engelbrecht *et al*., 2019), two delivered psychotherapy or counselling (de Menil *et al*., 2015; Engelbrecht *et al*., 2019), two offered support for income generation (de Menil *et al*., 2015; Asher *et al*., 2018) and one provided occupational therapy (art and music therapies) (Engelbrecht *et al*., 2019). Finally, traditional and faith-based healing was examined in one study: Gureje *et al*. (2020) examined collaborative care between traditional and faith healers and healthcare workers, but did not evaluate the impact of these healing components.

*Intervention delivery and settings*. Table 4 presents a summary of the delivery of psychosis interventions, by country. Six studies (Gohar *et al*., 2013; de Menil *et al*., 2015; Asher *et al*., 2018; Brooke-Sumner *et al*., 2018; Rami *et al*., 2018; Engelbrecht *et al*., 2019) included group-based components, either as the main mode of intervention delivery, or as one of the several components of an intervention. Four studies (Gohar *et al*., 2013; de Menil *et al*., 2015; Rami *et al*., 2018; Engelbrecht *et al*., 2019) used specialists, and five studies (de Menil *et al*., 2015; Asher *et al*., 2018; Brooke-Sumner *et al*., 2018; Gureje *et al*., 2020; Hanlon *et al*., 2020) used non-specialists in their delivery. The specialists employed included psychiatrists (Gohar *et al*., 2013; de Menil *et al*., 2015; Rami *et al*., 2018), and specialist occupational therapists (Engelbrecht *et al*., 2019). Non-specialists included healthcare workers and social workers not specializing in mental health (Brooke-Sumner *et al*., 2018, Gureje *et al*., 2020; Hanlon *et al*., 2020), and lay workers (de Menil *et al*., 2015; Asher *et al*., 2018). One study (Gureje *et al*., 2020) involved traditional and faith healers, and two (Sibeko *et al*., 2017; Thomas *et al*., 2017) utilized digital platforms in their delivery. A few studies involved carers, family or friends (Sibeko *et al*., 2017; Rami *et al*., 2018), or community members (Asher *et al*., 2018) as additional targets of the intervention, as opposed to delivery agents.

With respect to setting, six (de Menil *et al*., 2015; Sibeko *et al*., 2017; Thomas *et al*., 2017; Asher *et al*., 2018; Gureje *et al*., 2020; Hanlon *et al*., 2020) programmes were delivered in the community and six (Gohar *et al*., 2013; de Menil *et al*., 2015; Thomas *et al*., 2017; Brooke-Sumner *et al*., 2018; Engelbrecht *et al*., 2019; Hanlon *et al*., 2020) in facilities (these categories were not mutually exclusive, as many had facility- and community-based components). In one study (Rami *et al*., 2018), setting was not clear.

*Outcomes.* Outcomes and outcome measures are summarized in Table 5. Interventions targeted a range of outcomes, including psychiatric symptoms, cognition, service/treatment utilization, disability and functioning, quality of life and needs, stigma, discrimination and rights infringements, substance use and caregiver burden, although the vast majority were focused on symptom reduction and functional improvement. By far the most commonly used measure was the Positive and Negative Syndrome Scale (PANSS), which was applied in half of all studies. This was followed by the Brief Psychiatric Rating Scale (BPRS), which was used in three studies. Reductions in harmful practices like chaining were noted in two studies (Gureje *et al*., 2020; Hanlon *et al*., 2020), and stigma and discrimination in four (Asher *et al*., 2018; Brooke-Sumner *et al*., 2018; Gureje *et al*., 2020; Hanlon *et al*., 2020).

*Intervention impact*. Eight of the 10 included studies reported significant, positive effects of the interventions under study (Gohar *et al*., 2013; de Menil *et al*., 2015; Thomas *et al*., 2017; Brooke-Sumner *et al*., 2018; Rami *et al*., 2018; Engelbrecht *et al*., 2019; Gureje *et al*., 2020; Hanlon *et al*., 2020). Of the studies which did not report significant findings, one reported a null effect (Sibeko *et al*., 2017), while the other was a pilot study that was not powered to test for significance (Asher *et al*., 2018).

Interventions that resulted in improved symptoms included a district-level plan for task-shared mental health, manualized collaborative care by traditional and faith healers and primary healthcare workers, a family psychoeducation intervention and a community-based intervention with clinical and group components. Manualized collaborative care by traditional and faith healers and primary healthcare workers, occupational therapy and traditional healing interventions all resulted in reduced rates and/or duration of admission. An SMS text reminder of clinic appointments improved attendance, and a family psychoeducation intervention improved attitudes towards medication. A district-level plan for task-shared mental health reduced rates of substance use.

A cognitive skills training intervention improved some dimensions of cognitive function but did not produce significant improvements in other domains of cognition, nor in symptoms. Similarly, a non-specialist psychosocial intervention with treatment partners, psychoeducation and clinic appointment reminder (Sibeko *et al*., 2017) did not have a significant effect on symptoms or functioning.

Finally, in terms of outcomes related to participation in society and social inclusion, a non-specialist psychosocial intervention and manualized collaborative care by traditional and faith healers and primary healthcare workers reduced internalized stigma and improved rates of work, though the latter did not increase independent living or reduce experiences of victimization. A family psychoeducation intervention improved quality of life, and both this intervention and a district-level plan for task-shared mental health improved social functioning.

Although significance tests were not conducted, it is worth also noting that the CBR pilot attached to task-shared mental health care reported improvements in disability, symptoms, experiences of stigma, substance use and caregiver burden.

## Discussion

The broad search strategy and inclusion criteria employed in this review identified a range of interventions targeting different outcomes of psychotic conditions (mainly schizophrenia) across the African region.

Encouragingly, eight of the 10 included studies reported significant, positive effects of the interventions under study. Significant findings were reported for participants across a range of outcomes (including symptoms, cognition, service/treatment utilization, disability and functioning, quality of life and needs, stigma, discrimination and rights infringements, substance use and caregiver burden) and after receiving a number of different interventions (including education, awareness and social support initiatives, psychological and psychosocial programmes, clinical interventions and traditional and faith-based healing). However, we present these findings with the caveat that confidence in study findings was low to medium. Furthermore, while the outcomes and measurement tools selected to evaluate psychosis interventions was a topic of interest for this review, their validity for use in African populations was rarely discussed.

Psychoeducation and clinical interventions (such as clinical monitoring) were the most common. Despite the fact that most interventions were multicomponent, and even though we excluded purely pharmacological interventions, the studies identified in this review reflected a psychological and clinical focus. This was also mirrored in the reporting of outcomes, with symptom-based outcome measures used most frequently. Symptoms may arguably be easier to measure using standardized tools than are other outcomes. However, choice of outcome measure may also reflect programmes' focus on symptomatology over other domains. It may be useful for future studies to involve people with lived experience in study design to ensure that the measures used capture valued outcomes. While some programmes may have involved people with lived experience in their design or development, this was not commonly reported.

Regarding delivery agents for interventions, it is notable that a mix of professional and para-professional staff was employed. While task-shifting is certainly important, and this importance is evidenced by the involvement of non-specialists in many of the included studies, it is also noteworthy that in many cases mental health specialists were engaged in the delivery of psychosocial interventions. This may indicate investment in specialized care. Unfortunately, it was not possible in most publications to distinguish stand-alone interventions from those which were integrated within existing public sector services.

Overall, findings of this review would suggest that current practices do not yet reflect a fully holistic approach to intervention for people with psychosocial disabilities associated with psychosis in Africa. For instance, there is a substantial body of literature documenting the relationship between economic deprivation and psychosis (Brown *et al*., 2000; Burns and Esterhuizen, 2008; Read, 2010). Yet, social protection programming, cash transfers, microfinance and other interventions targeting poverty, were largely absent from the included studies. However, it is also possible that these practices exist, but have not yet been evaluated or documented in the peer-reviewed academic literature. For instance, the CBR guidelines developed by the WHO, International Labour Organization, United Nations Educational, Scientific and Cultural Organization and the International Disability and Development Consortium include examples of CBR for mental health and emphasize the importance of developing or strengthening community-based programmes across a range of domains, including health, education, livelihoods and social inclusion and empowerment (Hartley *et al*., 2009; World Health Organization, 2010). More recently, the WHO's Guidance on Community Mental Health Services has highlighted examples of good practice that promote rights and recovery, many of which have never been formally evaluated (WHO, 2021). We are also aware of a number of relevant study protocols and formative research studies that were returned by our search, but have not yet published findings, suggesting this could be a growing area of research (Ryan *et al*., 2019; Moran *et al*., 2020; Moro *et al*., 2022).

Nevertheless, the apparent focus on psychological and clinical programming and outcomes in this review does indicate a missed opportunity to address the challenges faced by people with psychosocial impairments, including people with severe mental health conditions like psychosis, in the evidence base to-date. People with psychotic conditions are more likely to have lower educational attainment (Rajji *et al*., 2013), higher unemployment rates (Marwaha and Johnson, 2004; Ramsay *et al*., 2012) and face social exclusion (Perry *et al*., 2011; Mfoafo-M'Carthy and Sossou, 2017; Lincoln *et al*., 2021), suggesting a real need for programming focused on a wide range of outcomes. It is notable, however, that this review does include studies presenting evidence of interventions that significantly reduced rates of internalized stigma and improved rates of engagement in work.

The studies identified by this review also reflect limited targeting of affected individuals' families (four studies out of 12), which is at odds with the pivotal role that familial carers play in supporting people with psychosis (Szmukler *et al*., 2003), particularly in African settings (Ohaeri, 2001; Fekadu, 2020; Yerriah *et al*., 2021). Another noteworthy point regarding targeting concerns participant age. Indeed, the age range of many of the included studies was very broad, and within studies with very broad age ranges, findings were not disaggregated by participant age. We did not find evidence that youth were targeted by interventions. Furthermore, how they are affected within broader programmes for a range of age groups, and whether these effects are differential to those of adults, remains unclear given the lack of disaggregation.

In terms of delivery, it is encouraging that an equal number of studies relied on specialist mental healthcare providers when compared to those involving non-specialist providers. This may reflect efforts to build capacity among non-specialists as part of a broader shift towards decentralization and task-shifting, reducing reliance on tertiary mental health and the scarce human resources in specialties. While interventions typically had a psychological or clinical focus, intervention delivery was not restricted to clinicians. We also identified an innovative example of collaboration between allopathic and non-allopathic care providers, among many other examples of non-specialist task-sharing.

Finally, in terms of setting, we noted an equal distribution between community and institutional delivery. This may also reflect efforts to deliver services to people with psychosocial disabilities in the communities where they live, as enshrined in Article 19 of the United Nations Convention on the Rights of Persons with Disabilities (Assembly, 2007). However, in the low-resource settings in which these studies were conducted, it is noteworthy that so few utilized digital platforms. While there may be need for caution in employing these approaches, given limited access to technologies in many LMICs and the barriers faced by people with psychosocial disabilities in particular, there may also be potential for digital interventions to help overcome challenges of service delivery in resource-constrained settings (Mpango *et al*., 2020). This may be an area where further intervention development and evaluation is needed.

### Limitations

As anticipated in our protocol, the heterogeneity of the included studies precludes any sort of meta-analysis that might help to quantify the impact of psychosis interventions in Africa. Evidence of impact should be interpreted with caution, particularly given the low-to-medium confidence in study findings reported by reviewers. Our confidence in study findings tool applies the weakest link in the chain principle, meaning that a study received an overall rating which was equal to their lowest rating on any single item. This can skew the overall appraisal of quality in the direction of low confidence. However, the tool used is pared-down compared to more common quality assessment tools for evidence synthesis, as it presents only the most essential elements of methodological and reporting rigour, and so the application of other tools would have been unlikely to alter this assessment. Perhaps, the most obvious limitation of this review is its restriction to English-language publications. Studies from Francophone and Lusophone Africa are notably absent, although some predominantly Arabic speaking countries like Egypt and Sudan are represented. Finally, this review should be updated in future to take into consideration new studies that may have been published in this fast-growing area of research since 2020, when the original search was conducted.

## Conclusion

This review identified a number of different interventions for adults and adolescents with psychosis in Africa, exploring not only their methods of evaluation and outcomes, but also how they are delivered and in what settings. Our main finding – that the peer-reviewed literature in this area is overwhelmingly focused on psychological and clinical interventions and outcomes – has important implications for practice. Namely, more attention to the diverse needs and priorities of people with psychosis is required in order to develop and evaluate holistic interventions that go beyond the health sector, in line with social models of psychosocial disability. Efforts should also be made to ensure that domains outside of health – including education, livelihoods, social inclusion and empowerment – are targeted by programming. There is a clear need to expand the evidence base whilst simultaneously ensuring that what is already shown to work for people with psychosis is implemented and scaled.

## Data Availability

The datasets used and/or analysed during the current study are available from the corresponding author on reasonable request.

## Supplementary material

For supplementary material accompanying this paper visit https://doi.org/10.1017/gmh.2022.25. click here to view supplementary material
