# Supplementary material for: Interventions for adolescents and adults with psychosis in Africa: a systematic review and narrative synthesis
Source: Glob Ment Health (Camb). 2022 May 27;9:223–40. doi: 10.1017/gmh.2022.25 (PMC9806991; doi:10.1017/gmh.2022.25)
Supplement: Supplementary file 1 [file S2054425122000255sup001.docx]

Supplementary Materials – Generic search strategy

**Population**. psychotic* OR schizoaffect* OR schizophren* OR (hear* n/2 voice*) OR paranoi* OR hallucinat* OR delusion* OR psychos* OR ‘sever*e mental*’ OR ‘serious* mental*

AND

**Location**. “Africa” OR “East Africa” OR “sub-Saharan Africa” OR “West Africa” OR “North Africa” OR Nigeria OR Ethiopia OR Egypt OR “Democratic Republic of the Congo” OR “South Africa” OR Tanzania OR Kenya OR Algeria OR Uganda OR Sudan OR Morocco OR Ghana OR Mozambique OR “Ivory Coast” OR Madagascar OR Angola OR Cameroon OR Niger OR “Burkina Faso” OR Mali OR Malawi OR Zambia OR Senegal OR Zimbabwe OR Chad OR Guinea OR Tunisia OR Rwanda OR “South Sudan” OR Benin OR Somalia OR Burundi OR Togo OR Libya OR “Sierra Leone” OR “Central African Republic” OR Eritrea OR “Republic of the Congo” OR Liberia OR Mauritania OR Gabon OR Namibia OR Botswana OR Lesotho OR “Equatorial Guinea” OR Gambia OR Guinea-Bissau OR Mauritius OR Swaziland OR Djibouti OR Reunion OR Comoros OR “Western Sahara” OR “Cape Verde” OR Mayotte OR “Sao Tome and Principe” OR Seychelles OR “Saint Helena, Ascension and Tristan da Cunha” OR eSwatini
